# Tetra­aqua­bis(3,5-di-4-pyridyl-1*H*-1,2,4-triazolido)cadmium(II) dihydrate

**DOI:** 10.1107/S1600536809024908

**Published:** 2009-07-11

**Authors:** Ti-Lou Liu, Yun-Liang Zhang

**Affiliations:** a Department of Pharmacy, Shaoyang Medical College, Shaoyang, Hunan 422000, People’s Republic of China

## Abstract

In the title compound, [Cd(C_12_H_8_N_5_)_2_(H_2_O)_4_]·2H_2_O, the Cd^II^ atom is located on an inversion center and is coordinated by the two N atoms [Cd—N = 2.278 (2) Å] and four O atoms [Cd—O = 2.304 (2)–2.322 (2) Å] in a distorted octa­hedral geometry. Inter­molecular O—H⋯O and O—H⋯N hydrogen bonds link the complex into a three-dimensional supra­molecular framework.

## Related literature

For the properties of hydrogen bonds in biological systems, see: Deisenhofer & Michel (1989 ▶). For extended supra­molecular structures, see: Beatty (2003 ▶); Li *et al.* (2006 ▶); Russell & Ward (1996 ▶). For comparitive bond distances, see: Wen *et al.* (2005 ▶); Fu *et al.* (2007 ▶).
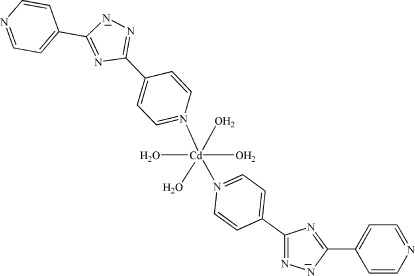


## Experimental

### 

#### Crystal data

[Cd(C_12_H_8_N_5_)_2_(H_2_O)_4_]·2H_2_O
*M* *_r_* = 664.97
Monoclinic, 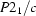

*a* = 7.5030 (15) Å
*b* = 15.748 (3) Å
*c* = 12.009 (2) Å
β = 106.68 (3)°
*V* = 1359.2 (5) Å^3^
*Z* = 2
Mo *K*α radiation
μ = 0.86 mm^−1^
*T* = 293 K
0.40 × 0.20 × 0.12 mm

#### Data collection

Bruker SMART CCD area-detector diffractometer
Absorption correction: multi-scan (*SADABS*; Bruker, 1998 ▶) *T*_min_ = 0.815, *T*_max_ = 0.911
9599 measured reflections
3101 independent reflections
2663 reflections with *I* > 2σ(*I*)
*R*_int_ = 0.035

#### Refinement

*R*[*F* ^2^ > 2σ(*F* ^2^)] = 0.030
*wR*(*F* ^2^) = 0.070
*S* = 1.01
3101 reflections
211 parameters
9 restraints
H atoms treated by a mixture of independent and constrained refinement
Δρ_max_ = 0.30 e Å^−3^
Δρ_min_ = −0.47 e Å^−3^

### 

Data collection: *SMART* (Bruker, 1998 ▶); cell refinement: *SAINT* (Bruker, 1998 ▶); data reduction: *SAINT*; program(s) used to solve structure: *SHELXS97* (Sheldrick, 2008 ▶); program(s) used to refine structure: *SHELXL97* (Sheldrick, 2008 ▶); molecular graphics: *SHELXTL* (Sheldrick, 2008 ▶); software used to prepare material for publication: *SHELXTL*.

## Supplementary Material

Crystal structure: contains datablocks global, I. DOI: 10.1107/S1600536809024908/bg2273sup1.cif

Structure factors: contains datablocks I. DOI: 10.1107/S1600536809024908/bg2273Isup2.hkl

Additional supplementary materials:  crystallographic information; 3D view; checkCIF report

## Figures and Tables

**Table 1:** Hydrogen-bond geometry (Å, °)

*D*—H⋯*A*	*D*—H	H⋯*A*	*D*⋯*A*	*D*—H⋯*A*
O1—H1*A*⋯N2^i^	0.84 (2)	1.95 (3)	2.768 (3)	164 (3)
O1—H1*B*⋯O3	0.85 (3)	1.95 (3)	2.786 (3)	169 (3)
O2—H2*A*⋯N3^ii^	0.85 (3)	1.98 (3)	2.829 (3)	171 (3)
O2—H2*B*⋯O3^iii^	0.85 (3)	1.95 (3)	2.758 (3)	161 (3)
O3—H3*A*⋯N4^iv^	0.85 (3)	2.06 (2)	2.895 (3)	171 (3)
O3—H3*B*⋯N5^v^	0.85 (3)	1.95 (2)	2.796 (3)	170 (3)

Symmetry codes: (i) 
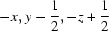
; (ii) 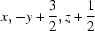; (iii) 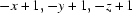; (iv) 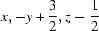; (v) 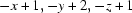.

**Fig. 1.**
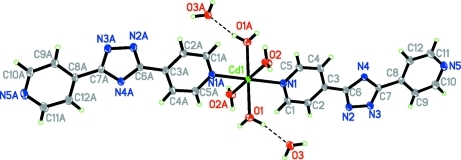
A view of the molecular structure of (I) with the atom-numbering scheme and 30% displacement ellipsoids. Atoms with the suffix A are generated by the symmetry operation (-x, -y + 1, -z + 1).

**Fig. 2.**
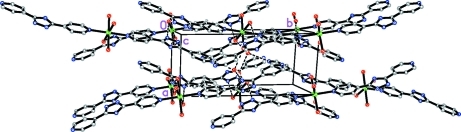
The 3-D network of (I).

## supplementary crystallographic information

### Comment

The hydrogen bond interaction plays a important role in some biological systems (Deisenhofer & Michel, 1989). Supramolecular assembly through hydrogen bonds has been extensively exploited to generate extended one-, two- and three-dimensional structures (Beatty *et al.*, 2003; Li *et al.*, 2006; Russell & Ward, 1996). As part of this ongoing work, We present here the synthesis and structural characterization of the title cadmium complex, [Cd(C_12_H_8_N_5_)_2_(H_2_O)_4_].2H_2_O, (I).

The molecule of the title complex, (Fig. 1), is centrosymmetric, so pairs of equivalent ligands lie *trans* to each other in a slightly distorted octahedral coordination geometry, *cis* angles deviating from 90° by less than 4°. with Cd—O bond length in the range 2.304 (2)–2.322 (2) Å and a Cd—N bond length of 2.278 (2) Å. These bond distances compare well with those in the literature (Wen *et al.*, 2005; Fu *et al.*, 2007). Molecules are linked by O—H···O and O—H···N hydrogen bonds (Table 1 and Fig. 2).

### Experimental

Cd(NO_3_)_2_.4H_2_O (0.5 mmol, 0.154 g), 1*H*-3,5-di(4-pyridyl)-1,2,4-triazole (0.5 mmol, 0.112 g), and water (12 ml) were placed in a 23-ml Teflon-lined Parr bomb. The bomb was heated at 453 K for 3 d. The colourless block-shapped crystals were filtered off and washed with water and acetone (yield 45%, based on Cd).

### Refinement

Hydrogen atoms of water molecules were located in a difference Fourier map and refined with distance restraints of O—H = 0.85 (2) Å and H···H = 1.39 (2) Å, and free isotropic U's. H atoms on C atoms were positoned geometrically and refined using a riding model, with C—H = 0.97 Å and *U*_iso_(H) = 1.2*U*_eq_(C).

### Crystal data

**Table:**

[Cd(C_12_H_8_N_5_)_2_(H_2_O)_4_]·2H_2_O	*F*(000) = 676
*M**_r_* = 664.97	*D*_x_ = 1.625 Mg m^−^^3^
Monoclinic, *P*2_1_/*c*	Mo *K*α radiation, λ = 0.71073 Å
Hall symbol: -P 2ybc	Cell parameters from 2567 reflections
*a* = 7.5030 (15) Å	θ = 2.6–27.3°
*b* = 15.748 (3) Å	µ = 0.86 mm^−^^1^
*c* = 12.009 (2) Å	*T* = 293 K
β = 106.68 (3)°	Block, colorless
*V* = 1359.2 (5) Å^3^	0.40 × 0.20 × 0.12 mm
*Z* = 2	

### Data collection

**Table:**

Bruker SMART CCD area-detector diffractometer	3101 independent reflections
Radiation source: fine-focus sealed tube	2663 reflections with *I* > 2σ(*I*)
graphite	*R*_int_ = 0.035
φ and ω scans	θ_max_ = 27.5°, θ_min_ = 3.1°
Absorption correction: multi-scan (*SADABS*; Bruker, 1998)	*h* = −9→9
*T*_min_ = 0.815, *T*_max_ = 0.911	*k* = −20→20
9599 measured reflections	*l* = −15→15

### Refinement

**Table:**

Refinement on *F*^2^	Primary atom site location: structure-invariant direct methods
Least-squares matrix: full	Secondary atom site location: difference Fourier map
*R*[*F*^2^ > 2σ(*F*^2^)] = 0.030	Hydrogen site location: inferred from neighbouring sites
*wR*(*F*^2^) = 0.070	H atoms treated by a mixture of independent and constrained refinement
*S* = 1.01	*w* = 1/[σ^2^(*F*_o_^2^) + (0.0271*P*)^2^ + *P*] where *P* = (*F*_o_^2^ + 2*F*_c_^2^)/3
3101 reflections	(Δ/σ)_max_ < 0.001
211 parameters	Δρ_max_ = 0.30 e Å^−^^3^
9 restraints	Δρ_min_ = −0.47 e Å^−^^3^

### Special details

**Table:**

Geometry. All e.s.d.'s (except the e.s.d. in the dihedral angle between two l.s. planes) are estimated using the full covariance matrix. The cell e.s.d.'s are taken into account individually in the estimation of e.s.d.'s in distances, angles and torsion angles; correlations between e.s.d.'s in cell parameters are only used when they are defined by crystal symmetry. An approximate (isotropic) treatment of cell e.s.d.'s is used for estimating e.s.d.'s involving l.s. planes.
Refinement. Refinement of *F*^2^ against ALL reflections. The weighted *R*-factor *wR* and goodness of fit *S* are based on *F*^2^, conventional *R*-factors *R* are based on *F*, with *F* set to zero for negative *F*^2^. The threshold expression of *F*^2^ > σ(*F*^2^) is used only for calculating *R*-factors(gt) *etc*. and is not relevant to the choice of reflections for refinement. *R*-factors based on *F*^2^ are statistically about twice as large as those based on *F*, and *R*- factors based on ALL data will be even larger.

### Fractional atomic coordinates and isotropic or equivalent isotropic displacement parameters (Å^2^)

**Table:**

	*x*	*y*	*z*	*U*_iso_*/*U*_eq_	
Cd1	0.0000	0.5000	0.5000	0.02624 (8)	
C1	0.0093 (4)	0.67991 (14)	0.3780 (2)	0.0362 (6)	
H1	−0.0392	0.6476	0.3113	0.043*	
C2	0.0355 (4)	0.76513 (14)	0.3660 (2)	0.0350 (5)	
H2	0.0038	0.7894	0.2923	0.042*	
C3	0.1089 (3)	0.81536 (13)	0.46302 (19)	0.0260 (5)	
C4	0.1464 (4)	0.77542 (14)	0.5700 (2)	0.0371 (6)	
H4	0.1919	0.8065	0.6382	0.045*	
C5	0.1161 (4)	0.68949 (15)	0.5749 (2)	0.0380 (6)	
H5	0.1430	0.6638	0.6476	0.046*	
C6	0.1502 (3)	0.90502 (13)	0.45109 (18)	0.0265 (5)	
C7	0.2512 (3)	1.02895 (14)	0.4805 (2)	0.0274 (5)	
C8	0.3340 (3)	1.10850 (13)	0.53511 (19)	0.0276 (5)	
C9	0.3496 (4)	1.17874 (16)	0.4689 (2)	0.0472 (7)	
H9	0.3127	1.1754	0.3882	0.057*	
C10	0.4202 (5)	1.25321 (16)	0.5236 (2)	0.0515 (8)	
H10	0.4285	1.2995	0.4772	0.062*	
C11	0.4620 (4)	1.19586 (16)	0.7006 (2)	0.0423 (6)	
H11	0.5012	1.2010	0.7811	0.051*	
C12	0.3919 (4)	1.11875 (15)	0.6541 (2)	0.0376 (6)	
H12	0.3837	1.0738	0.7027	0.045*	
H1A	0.081 (4)	0.4477 (19)	0.303 (2)	0.065 (11)*	
H2A	0.251 (5)	0.487 (2)	0.722 (2)	0.076 (12)*	
H3A	0.333 (4)	0.5708 (17)	0.2136 (15)	0.057 (10)*	
H1B	0.225 (4)	0.5023 (14)	0.349 (3)	0.050 (9)*	
H2B	0.371 (3)	0.474 (2)	0.656 (3)	0.071 (11)*	
H3B	0.419 (4)	0.6266 (12)	0.301 (2)	0.059 (9)*	
N1	0.0504 (3)	0.64121 (11)	0.48107 (16)	0.0309 (4)	
N2	0.1075 (3)	0.94209 (12)	0.34725 (16)	0.0338 (5)	
N3	0.1745 (3)	1.02282 (12)	0.36636 (17)	0.0351 (5)	
N4	0.2401 (3)	0.95673 (11)	0.53920 (16)	0.0269 (4)	
N5	0.4772 (3)	1.26344 (13)	0.6379 (2)	0.0413 (5)	
O1	0.1563 (3)	0.46448 (12)	0.36541 (15)	0.0358 (4)	
O2	0.2657 (3)	0.49379 (12)	0.65460 (16)	0.0416 (4)	
O3	0.3821 (3)	0.57563 (10)	0.28626 (15)	0.0349 (4)	

### Atomic displacement parameters (Å^2^)

**Table:**

	*U*^11^	*U*^22^	*U*^33^	*U*^12^	*U*^13^	*U*^23^
Cd1	0.03522 (14)	0.01796 (11)	0.02429 (13)	−0.00424 (9)	0.00655 (10)	−0.00067 (8)
C1	0.0537 (16)	0.0256 (11)	0.0261 (12)	−0.0094 (11)	0.0063 (11)	−0.0044 (9)
C2	0.0516 (15)	0.0269 (11)	0.0240 (11)	−0.0059 (11)	0.0067 (11)	0.0028 (9)
C3	0.0296 (11)	0.0208 (10)	0.0275 (11)	−0.0013 (8)	0.0079 (9)	0.0014 (8)
C4	0.0577 (16)	0.0233 (11)	0.0255 (12)	−0.0090 (11)	0.0042 (12)	−0.0027 (9)
C5	0.0596 (17)	0.0254 (11)	0.0249 (12)	−0.0073 (11)	0.0057 (12)	0.0031 (9)
C6	0.0331 (12)	0.0206 (10)	0.0241 (11)	−0.0013 (9)	0.0056 (9)	0.0011 (8)
C7	0.0333 (12)	0.0205 (9)	0.0295 (12)	−0.0024 (9)	0.0106 (10)	0.0006 (9)
C8	0.0302 (12)	0.0204 (10)	0.0314 (12)	−0.0019 (9)	0.0075 (10)	−0.0006 (9)
C9	0.078 (2)	0.0311 (13)	0.0308 (13)	−0.0176 (13)	0.0137 (14)	−0.0018 (10)
C10	0.084 (2)	0.0276 (12)	0.0446 (16)	−0.0188 (14)	0.0214 (16)	0.0009 (11)
C11	0.0532 (16)	0.0364 (13)	0.0303 (13)	−0.0066 (12)	0.0010 (12)	−0.0046 (10)
C12	0.0496 (15)	0.0260 (11)	0.0327 (13)	−0.0042 (10)	0.0046 (12)	0.0042 (9)
N1	0.0435 (11)	0.0199 (9)	0.0278 (10)	−0.0049 (8)	0.0077 (9)	0.0005 (7)
N2	0.0487 (13)	0.0241 (9)	0.0258 (10)	−0.0087 (9)	0.0066 (9)	0.0016 (7)
N3	0.0523 (13)	0.0239 (9)	0.0265 (10)	−0.0089 (9)	0.0073 (10)	0.0020 (8)
N4	0.0348 (10)	0.0192 (9)	0.0261 (10)	−0.0021 (7)	0.0078 (8)	0.0005 (7)
N5	0.0488 (13)	0.0280 (10)	0.0458 (13)	−0.0092 (9)	0.0112 (11)	−0.0087 (9)
O1	0.0405 (10)	0.0387 (10)	0.0295 (9)	−0.0055 (8)	0.0120 (8)	−0.0026 (8)
O2	0.0349 (10)	0.0577 (12)	0.0288 (9)	0.0058 (9)	0.0035 (8)	−0.0038 (8)
O3	0.0458 (10)	0.0258 (8)	0.0299 (9)	−0.0037 (7)	0.0060 (8)	−0.0015 (7)

### Geometric parameters (Å, °)

**Table:**

Cd1—N1^i^	2.278 (2)	C7—N4	1.353 (3)
Cd1—N1	2.278 (2)	C7—C8	1.467 (3)
Cd1—O2	2.304 (2)	C8—C12	1.379 (3)
Cd1—O2^i^	2.304 (2)	C8—C9	1.386 (3)
Cd1—O1^i^	2.322 (2)	C9—C10	1.373 (3)
Cd1—O1	2.322 (2)	C9—H9	0.9300
C1—N1	1.334 (3)	C10—N5	1.325 (3)
C1—C2	1.370 (3)	C10—H10	0.9300
C1—H1	0.9300	C11—N5	1.327 (3)
C2—C3	1.385 (3)	C11—C12	1.376 (3)
C2—H2	0.9300	C11—H11	0.9300
C3—C4	1.385 (3)	C12—H12	0.9300
C3—C6	1.461 (3)	N2—N3	1.362 (3)
C4—C5	1.376 (3)	O1—H1A	0.844 (17)
C4—H4	0.9300	O1—H1B	0.845 (17)
C5—N1	1.331 (3)	O2—H2A	0.857 (17)
C5—H5	0.9300	O2—H2B	0.847 (17)
C6—N2	1.330 (3)	O3—H3A	0.848 (16)
C6—N4	1.352 (3)	O3—H3B	0.850 (17)
C7—N3	1.329 (3)		
			
N1^i^—Cd1—N1	180.0	N3—C7—N4	113.8 (2)
N1^i^—Cd1—O2	90.47 (7)	N3—C7—C8	121.7 (2)
N1—Cd1—O2	89.53 (7)	N4—C7—C8	124.5 (2)
N1^i^—Cd1—O2^i^	89.53 (7)	C12—C8—C9	116.6 (2)
N1—Cd1—O2^i^	90.47 (7)	C12—C8—C7	122.1 (2)
O2—Cd1—O2^i^	180.0	C9—C8—C7	121.3 (2)
N1^i^—Cd1—O1^i^	91.96 (7)	C10—C9—C8	119.5 (2)
N1—Cd1—O1^i^	88.04 (7)	C10—C9—H9	120.3
O2—Cd1—O1^i^	86.71 (7)	C8—C9—H9	120.3
O2^i^—Cd1—O1^i^	93.29 (7)	N5—C10—C9	124.3 (2)
N1^i^—Cd1—O1	88.04 (7)	N5—C10—H10	117.8
N1—Cd1—O1	91.96 (7)	C9—C10—H10	117.8
O2—Cd1—O1	93.29 (7)	N5—C11—C12	124.3 (2)
O2^i^—Cd1—O1	86.71 (7)	N5—C11—H11	117.9
O1^i^—Cd1—O1	180.0	C12—C11—H11	117.9
N1—C1—C2	122.9 (2)	C11—C12—C8	119.6 (2)
N1—C1—H1	118.5	C11—C12—H12	120.2
C2—C1—H1	118.5	C8—C12—H12	120.2
C1—C2—C3	120.4 (2)	C5—N1—C1	117.07 (19)
C1—C2—H2	119.8	C5—N1—Cd1	120.33 (15)
C3—C2—H2	119.8	C1—N1—Cd1	122.54 (15)
C2—C3—C4	116.5 (2)	C6—N2—N3	105.89 (18)
C2—C3—C6	120.91 (19)	C7—N3—N2	105.73 (18)
C4—C3—C6	122.5 (2)	C6—N4—C7	100.94 (18)
C5—C4—C3	119.6 (2)	C10—N5—C11	115.8 (2)
C5—C4—H4	120.2	Cd1—O1—H1A	111 (2)
C3—C4—H4	120.2	Cd1—O1—H1B	117 (2)
N1—C5—C4	123.5 (2)	H1A—O1—H1B	108 (2)
N1—C5—H5	118.3	Cd1—O2—H2A	117 (2)
C4—C5—H5	118.3	Cd1—O2—H2B	128 (2)
N2—C6—N4	113.65 (19)	H2A—O2—H2B	108 (2)
N2—C6—C3	121.01 (19)	H3A—O3—H3B	108 (2)
N4—C6—C3	125.27 (19)		
			
N1—C1—C2—C3	0.6 (4)	C2—C1—N1—C5	1.3 (4)
C1—C2—C3—C4	−2.3 (4)	C2—C1—N1—Cd1	178.6 (2)
C1—C2—C3—C6	175.5 (2)	O2—Cd1—N1—C5	−38.2 (2)
C2—C3—C4—C5	2.2 (4)	O2^i^—Cd1—N1—C5	141.8 (2)
C6—C3—C4—C5	−175.5 (2)	O1^i^—Cd1—N1—C5	48.5 (2)
C3—C4—C5—N1	−0.4 (4)	O1—Cd1—N1—C5	−131.5 (2)
C2—C3—C6—N2	4.4 (3)	O2—Cd1—N1—C1	144.6 (2)
C4—C3—C6—N2	−178.0 (2)	O2^i^—Cd1—N1—C1	−35.4 (2)
C2—C3—C6—N4	−172.4 (2)	O1^i^—Cd1—N1—C1	−128.6 (2)
C4—C3—C6—N4	5.2 (4)	O1—Cd1—N1—C1	51.4 (2)
N3—C7—C8—C12	171.4 (2)	N4—C6—N2—N3	0.9 (3)
N4—C7—C8—C12	−6.7 (4)	C3—C6—N2—N3	−176.3 (2)
N3—C7—C8—C9	−5.4 (4)	N4—C7—N3—N2	0.3 (3)
N4—C7—C8—C9	176.4 (2)	C8—C7—N3—N2	−178.0 (2)
C12—C8—C9—C10	0.0 (4)	C6—N2—N3—C7	−0.6 (3)
C7—C8—C9—C10	177.0 (3)	N2—C6—N4—C7	−0.7 (3)
C8—C9—C10—N5	0.4 (5)	C3—C6—N4—C7	176.3 (2)
N5—C11—C12—C8	0.6 (4)	N3—C7—N4—C6	0.2 (3)
C9—C8—C12—C11	−0.4 (4)	C8—C7—N4—C6	178.5 (2)
C7—C8—C12—C11	−177.5 (2)	C9—C10—N5—C11	−0.3 (5)
C4—C5—N1—C1	−1.4 (4)	C12—C11—N5—C10	−0.2 (4)
C4—C5—N1—Cd1	−178.7 (2)		

Symmetry codes: (i) −*x*, −*y*+1, −*z*+1.

### Hydrogen-bond geometry (Å, °)

**Table:**

*D*—H···*A*	*D*—H	H···*A*	*D*···*A*	*D*—H···*A*
O1—H1A···N2^ii^	0.84 (2)	1.95 (3)	2.768 (3)	164 (3)
O1—H1B···O3	0.85 (3)	1.95 (3)	2.786 (3)	169 (3)
O2—H2A···N3^iii^	0.85 (3)	1.98 (3)	2.829 (3)	171 (3)
O2—H2B···O3^iv^	0.85 (3)	1.95 (3)	2.758 (3)	161 (3)
O3—H3A···N4^v^	0.85 (3)	2.06 (2)	2.895 (3)	171 (3)
O3—H3B···N5^vi^	0.85 (3)	1.95 (2)	2.796 (3)	170 (3)

Symmetry codes: (ii) −*x*, *y*−1/2, −*z*+1/2;  (iii) *x*, −*y*+3/2, *z*+1/2; (iv) −*x*+1, −*y*+1, −*z*+1; (v) *x*, −*y*+3/2, *z*−1/2; (vi) −*x*+1, −*y*+2, −*z*+1.
